# Metagenomic Approaches to Analyze Antimicrobial Resistance: An Overview

**DOI:** 10.3389/fgene.2020.575592

**Published:** 2021-01-18

**Authors:** Vinicius A. C. de Abreu, José Perdigão, Sintia Almeida

**Affiliations:** ^1^Laboratório de Bioinformática e Computação de Alto Desempenho (LaBioCad), Faculdade de Computação (FACOMP), Universidade Federal do Pará, Belém, Brazil; ^2^Central de Genômica e Bioinformática (CeGenBio), Núcleo de Pesquisa e Desenvolvimento de Medicamentos (NPDM), Departamento de Fisiologia e Farmacologia, Universidade Federal do Ceará, Fortaleza, Brazil

**Keywords:** antimicrobial resistance genes, horizontal gene transfer, metagenomic analysis, resistome, Shotgun metagenome sequencing, database

## Abstract

Antimicrobial resistance is a major global public health problem, which develops when pathogens acquire antimicrobial resistance genes (ARGs), primarily through genetic recombination between commensal and pathogenic microbes. The resistome is a collection of all ARGs. In microorganisms, the primary method of ARG acquisition is horizontal gene transfer (HGT). Thus, understanding and identifying HGTs, can provide insight into the mechanisms of antimicrobial resistance transmission and dissemination. The use of high-throughput sequencing technologies has made the analysis of ARG sequences feasible and accessible. In particular, the metagenomic approach has facilitated the identification of community-based antimicrobial resistance. This approach is useful, as it allows access to the genomic data in an environmental sample without the need to isolate and culture microorganisms prior to analysis. Here, we aimed to reflect on the challenges of analyzing metagenomic data in the three main approaches for studying antimicrobial resistance: (i) analysis of microbial diversity, (ii) functional gene analysis, and (iii) searching the most complete and pertinent resistome databases.

## Introduction

Bacterial resistance, which is closely associated with the use of antimicrobial agents, is considered one of the most persistent global public health problems (Enne and Bennett, 2010; Giedraitienė et al., 2011). However, it is not a new phenomenon. Resistance to penicillin developed in the 1940s, immediately after the large-scale use of the antibiotic. Healthcare was the first field to face challenges created by the indiscriminate use of antibiotics. However, medicine is not alone, and the fields of agriculture, livestock farming, and aquaculture are also being affected by the increasing, continued use of antibiotics, which drives the selection of resistant bacterial populations in environments and contributes to antimicrobial resistance (Barbosa and Levy, 2000; Van Boeckel et al., 2015; von Wintersdorff et al., 2016).

Antimicrobial resistance (Table 1) develops when pathogens acquire antimicrobial resistance genes (ARGs). The acquisition of ARGs primarily occurs through genetic recombination between commensal and pathogenic microbes and is associated with the conjugation mechanism of horizontal gene transfer (HGT) (Brown and Wright, 2016; Munita and Arias, 2016). Resistance is a mechanism naturally used by bacteria, whether induced or not induced. However, the large-scale use of antibiotics drives the rapid development of highly antimicrobial-resistant strains. Antibiotic resistance spreads through genetic material exchange, primarily between bacteria of the same genus, and, at a minor frequency, between phyla (von Wintersdorff et al., 2016; Wybouw et al., 2016), resulting in the development of potentially harmful bacteria.

**TABLE 1:** Resistance mechanisms, antibiotic resistance genes, and gene localization.

Chemical class	Target	Action	Genes	Gene localization	Reference(s)
Sulfonamides*	Folate synthesis	Bacteriostatic	*sulI, sulII*	P, T	Xu et al., 2018
β-Lactams	Cell-wall synthesis	Bactericidal	*ampC, blaTEM, qnrS, tetW*	P,C	Ferro et al., 2017; Pandey and Cascella, 2020
Amphenicols	Protein synthesis	Bacteriostatic Bactericidal	*fexA, cat, cmlA, floR, cfr, fex*	P, T, C	Rahal and Simberkoff, 1979; Kehrenberg and Schwarz, 2006; He et al., 2016
Aminoglycosides	Protein synthesis	Bactericidal	*rmtA, rmtB, armA, gar*	P	
Tetracyclines	Protein synthesis	Bacteriostatic Bactericidal	tet*	P, T	Rahal and Simberkoff, 1979; Roberts, 2005
Macrolides	Protein synthesis	Bacteriostatic	erm*, carA, ole*, smrB, tlrC, vgaA, vgaB, lmrA, mefA, msr*, lsaA, lsaB, ereA, ereB, vgbA, vgbB, inuA, inuB, vat*, mph*	P, T	Kanfer et al., 1998; Roberts, 2005
Glycopeptides	Cell-wall synthesis	Bactericidal	van*	P	Binda et al., 2014; Lebreton and Cattoir, 2019
Oxazolidinones	Protein synthesis	Bacteriostatic	*optrA, cfr*	P, C	Diekema and Jones, 2001; Wang et al., 2015
Ansamycins	RNA synthesis	Bactericidal	*rpoB*	P	Floss and Yu, 2005
Quinolones	DNA synthesis	Bactericidal	*qnr*	P	Heeb et al., 2011; Hernández et al., 2011
Streptogramins	Protein synthesis	Bactericidal	erm*, carA, ole*, smrB, tlrC, vgaA, vgaB, lmrA, mefA, msr*, lsaA, lsaB, ereA, ereB, vgbA, vgbB, inuA, inuB, vat*, mph*	P, T	Roberts, 2005
Lipopeptides	Protein synthesis	Bactericidal	*mprF, yycG, rpoB, rpoC, cls2, pgsA, agrA, prs, pnpA*	P, C	Montero et al., 2008; Gómez Casanova et al., 2017
Lincosamides	Protein synthesis	Bacteriostatic	erm*, carA, ole*, smrB, tlrC, vgaA, vgaB, lmrA, mefA, msr*, lsaA, lsaB, ereA, ereB, vgbA, vgbB, inuA, inuB, vat*, mph*	P, T	Tenson et al., 2003; Roberts, 2005
Phenicols	Protein synthesis	Bacteriostatic Bactericidal	*fexA, cat, cmlA, floR, cfr, fex*	P, T, C	Kehrenberg and Schwarz, 2006; He et al., 2016
Pyrimidines	DNA synthesis	Bactericidal	*dfrK, dfrD, dhfrI, dhfrX*	P	Sundstrom and Skold, 1990; Parsons et al., 1991; Charpentier and Courvalin, 1997; Petersen et al., 2000; Masters et al., 2003; Kadlec and Schwarz, 2009
Sulfonamides	DNA Synthesis	Bactericidal	*sul*(1-4)	P, T	Connor, 1998; Razavi et al., 2017
Rifamycins	RNA Synthesis	Bactericidal	*rpoB*	P	Floss and Yu, 2005
Lipopeptides	Protein Synthesis	Bactericidal	*mprF, yycG, rpoB, rpoC, cls2, pgsA, agrA, prs, pnpA, pmrHFIJKLM, pagP, phoP*	P, C	Thorne and Alder, 2002; Montero et al., 2008; Gómez Casanova et al., 2017
Cationic peptides	DNA synthesis, RNA synthesis, Protein synthesis, Cell-wall synthesis	Bactericidal	*pmrHFIJKLM, pagP, phoP*	C	Devine and Hancock, 2002; Hale and Hancock, 2007

P, plasmid; C, chromosome; T, transposon; *Tet gene family, *Erm gene family, *cat gene family, *fex gene family, *ole gene family, *msr gene family, *vat gene family, *mph gene family, *Van gene family.

Although numerous recent and ongoing research efforts have addressed bacterial virulence and multi-resistance mechanisms, the processes governing bacterial fitness, competition, dissemination, and adaptability remain poorly understood. Little is known about the diversity, distribution, and origin of resistance genes, especially those of most environmental bacteria that cannot be cultured under laboratory conditions (Schmieder and Edwards, 2012). The development, acquisition, and dissemination of ARGs are critical aspects of antimicrobial resistance, and the microbial community as a whole contributes to the generation of the antimicrobial resistome, rather than an individual ARG source organism (Bello-López et al., 2019; De, 2019). Therefore, understanding and identifying HGTs among pathogenic and non-pathogenic species may aid the determination of the mechanisms underlying resistance transmission and dissemination. The use of high-throughput sequencing technologies has made ARG sequence analyses feasible and accessible. Metagenomics, in particular, has facilitated the analysis of antimicrobial resistance in communities.

The term metagenomics, first used by Handelsman et al. (1998), originates from conventional microbial genomics and reflects the fact that pure cultures are not required for sequencing. The metagenomics approach is used to analyze the genomic data of environmental samples without the need to first isolate and culture microorganisms (Roh and Villatte, 2008; Cowan et al., 2015). Metagenomic analysis enables the prediction of new taxa (phyla, orders, genera, and candidate species) and genome reconstruction of organisms that cannot be cultured *in vitro*. The definition of community structures allows a deeper understanding of the relationships between individual components of a community and their dynamics in response to the selective pressure of a space-time parameter (Alves et al., 2018). Therefore, the metagenomic analysis of taxonomic (structural) assignment facilitates better identification of microbial communities, the discovery of new microbial metabolic capacities, and the inference of microbial functions in microbiomes where they inhabit (Simmons et al., 2014; Eloe-Fadrosh et al., 2016). Thus, sequence-based functional metagenomics is a powerful tool, widely used to discover resistance genes and identify and understand resistance mechanisms (Pehrsson et al., 2013; Xing et al., 2020). The robust structural and functional aspects of metagenomic data aid the study of antibacterial resistance.

A series of pipelines and reviews have focused on describing the best platforms for metagenomic statistical analyses and benchmarking metrics (Bengtsson-Palme et al., 2017; Quince et al., 2017; Boolchandani et al., 2019; Tamames and Puente-Sánchez, 2019; Ye et al., 2019), but this is not our goal. In this review, we have focused on the three main approaches used for metagenomic analysis of antimicrobial resistance: (i) analysis of microbial diversity, (ii) functional gene analysis, and (iii) searching the most complete and relevant resistome databases available. We will also comment on the challenges related to analyzing metagenomic data.

## Metagenomic Analysis of Resistance Genes

For several years, pathogenic bacteria have been the focus of antibiotic resistance research. This line of research has facilitated the identification of critical mechanisms that mediate bacterial antibiotic resistance. Among the mechanisms of antibiotic resistance, the four most important are (McManus, 1997; Munita and Arias, 2016): (i) enzymatic modification or destruction of the antibiotic, which usually involves the overproduction of enzymes that inactivate the antibiotic (e.g., β-lactamases and aminoglycosides kinases), (ii) alteration of the antibiotic target molecule to reduce its binding capacity, (iii) modification of metabolic pathways and regulatory networks to circumvent the effect of the antibiotic, and (iv) reduction of the intracellular accumulation of the antibiotic by decreasing cellular permeability to it or activating efflux mechanisms to export the harmful molecule.

However, an increasing number of resistance studies have provided new insight into microbial pathogenicity by analyzing the ARGs of both pathogenic and non-pathogenic bacteria (Beceiro et al., 2013; Roberts, 2017). This work raised interest in the genomes of non-pathogenic organisms based on the knowledge that comparative genomic analysis might aid the elucidation of gene associations relevant to antimicrobial resistance and indicate the presence or absence of ARGs. Mass sequencing and complete genome analysis have contributed to important advances in our understanding of bacterial resistance, genes that confer this resistance, and other phenotypes of interest. Moreover, data obtained from genomic analyses have revealed the remarkable genetic plasticity of bacteria, which enables them to respond to a wide variety of threats, including antibiotics. However, to understand the functioning of sets of genes that can acquire antibiotic resistance in resistomes, metagenomic methods are increasingly being used (Ghosh et al., 2013; Costa et al., 2015; Wang et al., 2020; Zhao et al., 2020). Metagenomic approaches can be function- or sequence-based (Schloss and Handelsman, 2003). In sequence-based methods, multiple sequence reads are generated and analyzed using sequence analysis software.

The most comprehensive approach for metagenome sequencing is complete genome sequencing; this approach allows the study of the structural and functional diversities of a microbial community by identifying genes and metabolic pathways and reconstructing almost complete bacterial genomes (Chen and Pachter, 2005; De, 2019). The main advantage of this approach is its sensitivity, as it allows the detection of a greater abundance of species and identification of potential ARGs.

Complete metagenomic sequencing, since it was implemented, has had a tremendous impact on the study of structural and functional microbial diversities in environmental and clinical samples and has been an alternative to rRNA sequencing (Escobar-Zepeda et al., 2018). Alternatively, functional metagenomics employ different approaches to study genes of interest, including gene cloning and sequencing and biochemical analysis (Ngara and Zhang, 2018; Tamames et al., 2019). Functional metagenomics are mostly used for the identification of resistance genes.

However, some challenges affect the quality of metagenomic analysis, with the first being low sensitivity in detecting minority populations that harbor resistance genes, which has proved to be an obstacle at the time of analysis (Lynch and Neufeld, 2015). The second is the low specificity in identifying allelic variants, which can have substantial impact, as different variants can impart different phenotypic susceptibilities (Forslund et al., 2013). To overcome these challenges, metagenomic analyses must employ both sequence- and function-based approaches, including functional gene annotation (Chistoserdovai, 2010; Lam et al., 2015) in the analysis pipeline, and heterologous expression of identified genes (Tripathi and Nailwal, 2020).

## Taxonomic Assignment

Horizontal gene transfer is a common method of genetic transfer between species of the same genus or with similar characteristics (Soucy et al., 2015). Thus, studying taxonomic assignments of resistome elements is fundamental for identifying bacteria that shape a resistome. Indeed, the microbial community composition or relative abundance of sampled organisms can be inferred through the taxonomic assignment analysis of resistome elements (Ruppé et al., 2019; Rice et al., 2020). Identifying the bacterial community composition can be accomplished via two distinct approaches: (i) direct measurement of raw data, which does not require the assembly of contigs and (ii) the assembly of contigs for subsequent composition inference. Both strategies have weaknesses and strengths (Mathe, 2002).

Taxonomic classification without the assembly of contigs is a faster approach, with a lower computational cost and no assembly problems (Rodríguez-Brazzarola et al., 2018). However, the quality and length of sequences are important during taxonomic assignment analysis, and poor-quality or short sequences, which are common in the non-assembly based approach, tend to generate matches with low statistical significance (Breitwieser et al., 2019; Ye et al., 2019).

Contrarily, the length of contigs is an advantage for taxonomic classification using contig assembly. Thus, this approach predominantly makes use of databases (Rodríguez-Brazzarola et al., 2018). Moreover, in some cases, contig assembly may enable partial genome reconstruction of a previously unknown organism. However, chimeric contig formation is possible owing to sample heterogeneity, which can be related to sample origin, and sample and sequence quality. All these features are closely linked to assembly quality, which influences classification quality.

In ARG analyses, genome assembly can help differentiate between bacteria in terms of conserved regions like ribosomes, possible HGT regions, and several classes of transposable elements. This is because the reduced size of gene sequences directly impacts gene annotation transfer and studies of biological mechanisms associated with resistance. Thus, taxonomic assignment by contig assembly tends to better facilitate the identification and understanding of resistance mechanisms, such as the understanding of microbiota structural relationship roles in resistome studies. However, it is important to emphasize that researchers must be aware of the type of sample being worked with, if the sample is too heterogeneous and if there is sufficient computational power to analyze the amount of data collected. Even for good-quality, long sequences, taxonomic classification without assembly could be a more appropriate approach from a computational point of view, depending on the dataset and the computational power available (Rodríguez-Brazzarola et al., 2018).

Notably, studying the taxonomic assignments of resistome elements using high-throughput sequencing goes beyond identifying ARGs in host-pathogen relations and can be used to study resistomes in environmental samples, such as those from water reservoirs (Ekwanzala et al., 2020; Yu et al., 2020), hospitals (MetaSUB Consortium et al., 2020), livestock wastewater and feces (Jia S. et al., 2017), human feces (Karkman et al., 2019), soil (Chen et al., 2017), air (Yang et al., 2018; Li et al., 2021), and biogeographical and biogeochemical processes (Quinn et al., 2014; Kuang et al., 2016; Roose-Amsaleg and Laverman, 2016; Liu et al., 2018).

## Functional Characterization and Databases

Studying taxonomic signatures enables a better understanding of the relationships between the members of a microbial community. Alternatively, functional metagenomic approach aims to identify functions within the community via the discovery of new enzymes, groups of biosynthetic genes, and ARGs. The functional annotation of a metagenome is similar to its genomic annotation, such that predicted gene sequences are compared to existing sequences in annotated databases (Dong and Strous, 2019). Thus, the high-throughput sequencing of microbial community genomes is a powerful tool to generate information about gene functions, metabolic pathways, and microbial genome evolution (Zhang et al., 2011).

There is a wide range of databases and tools to classify the taxonomic profile of a community and performing functional analyses; thus, the choice of reference database can have important implications for the quality of information obtained. There are three important points regarding sequence- and function-based analyses. First, functional analysis provides an opportunity to perform various sub-analyses, depending on the sequencing depth, including functional category, protein family, gene ontology, protein–protein interaction, pathway, and subsystem analysis. Second, for both types of analysis methods, researchers can work with assembled or non-assembled data. Finally, there are tools, usually open source tools, such as QIIME (Caporaso et al., 2010), Mothur (Schloss et al., 2009), and MEGAN (Huson et al., 2007), that perform both types of analysis.

Genomic annotation employs sequence comparison with similarity-based search tools, such as BLAST+, which was developed by the National Center for Biotechnology Information (NCBI) (Altschul et al., 1997). DIAMOND (Buchfink et al., 2015) performs pairwise sequence alignment for protein and translated DNA searches, which are designed for the high performance analysis of large sequence data; it has the advantage of being fast and is, therefore, attractive for the annotation of huge volumes of metagenomic data. USEARCH (Edgar, 2010) offers search and grouping algorithms that are faster than BLAST. RAPSearch2 (Zhao et al., 2012) is similar to BLAST, in that it uses flexible-length seeds on a reduced amino acid alphabet of ten symbols with the differential. Tools, such as BLAST, offer their own dataset (NR and RefSeq are most used), whereas others offer only alignment options, requiring the use of a third-party dataset (nr/nt, RefSeq, Env_NR, and UniProt). In both cases, it is necessary to download datasets separately or create one’s own local dataset. These tools use their databases for annotation or allow the user to employ a third-party database.

Although there are good database options and tools for comprehensive metagenomic analyses, continuous improvement for the detection and characterization of genetic elements is necessary, as it is important for understanding resistance acquisition over time and evolutionary dynamics. Thus, resistome databases must be constantly updated to include newly identified variant sequences, inserts, and deletions to improve our understanding of these variations in context of resistance (Danko et al., 2019). Moreover, the use of a non-specific or generalist database could generate inherent database bias for the target niche or organism. The choice of an appropriate database for sequence annotation is essential. This choice should be based on the type of data and ecosystem studied. We have highlighted below, the most frequently cited specialist databases for ARGs that allow metagenomic data input (Table 2), including ResFinder, Comprehensive Antibiotic Resistance Database (CARD), MEGARes, ARG-database, and Resfams.

**TABLE 2:** Bioinformatic resources for studying ARGs identified using targeted metagenomics.

Name	DC	Website	Citation PubMed/Scholar	Reference(s)
RAC	yes	http://www2.chi.unsw.edu.au/rac	14/56	Tsafnat et al., 2011
MvirDB	Yes	http://mvirdb.llnl.gov/	93/195	Zhou et al., 2007
ARDB	Yes	http://ardb.cbcb.umd.edu/	385/872	Liu and Pop, 2009
ResFinder	No	https://cge.cbs.dtu.dk/services/ResFinder/	1,176/2,234	Zankari et al., 2013
CARD	No	https://card.mcmaster.ca/home	441/955	McArthur et al., 2013
MEGARes	No	https://megares.meglab.org/	70/145	Doster et al., 2019
ARG-database	No	https://smile.hku.hk/SARGs	34/106	Yang et al., 2016
Resfams	No	http://www.dantaslab.org/resfams	273/388	Floss and Yu, 2005

*ARG, Antimicrobial resistance gene; DC, Discontinued.*

ResFinder is one of the oldest databases that keeps its sequences up to date. It extracts information from other databases, such as the Lahey^1^ database and ARDB (both now defunct). ResFinder also sources information from published literature, including reviews (Zankari et al., 2013). It uses the BLAST algorithm to assess sequence similarity. Fully- or draft-assembled sequences from different platforms, genomes or metagenomes, and long or short reads can be used as inputs for ResFinder.

Comprehensive Antibiotic Resistance Database is based on the core components of antimicrobial resistance, including genes and proteins, and utilizes published literature and controlled terminology to robustly investigate data. It is the most commonly used database in metagenomic projects. In addition to having a curated database (Jia B. et al., 2017), it includes resistome data that were computationally predicted in continuation of the ARDB project, which is now defunct.

MEGARes, a database of approximately 8,000 manually curated resistance genes with hierarchical statistical analysis, was published in 2016 and updated in 2019 (Doster et al., 2019). It relies on a specific Galaxy pipeline, although it offers the alternative option of downloading the entire database for integration with custom pipelines. The MEGARes dataset comprises several sources, including the curated CARD database (Doster et al., 2019).

Antimicrobial resistance gene-database is hierarchically structured (ARG type-subtype-reference sequence). Its first version integrated ARGs from ARDB and CARD, and redundant sequences were removed. When it was updated in 2018 (Yin et al., 2018), proteins from the NCBI-NR database were added, thereby tripling the number of sequences in the first version. Based on a specific Galaxy pipeline, the latest version also offers the option to download the database, allowing the integration of the available data with a custom analysis pipeline.

Resfams is organized by ontology with a curated database of protein families and associated profile hidden Markov models (HMMs) and protein sequences from the CARD database, the Lactamase Engineering Database, and Jacoby and Bush’s collection of curated beta-lactamase proteins. It was designed to quantitatively understand the relationship between human and environmental resistomes, with an analysis of over 6000 microbial genomes. It was last updated in 2018 (Gibson et al., 2015).

Although the databases fully complement one another and are often redundant, they continue to be cited as having individual specificities for particular datasets, which hinders recommendations. Given the importance of studies on microbial resistance and the quality of data obtained, it is essential that a platform-independent dataset be available for the antibiotic resistance research community. In one sequence database (DNA/Protein/raw data sequences), INSDC (International Nucleotide Sequence Database), initiatives for the unification and integration have already been implemented. INSDC is a standardization and unification initiative among the main sequence databases (DDBJ, EMBL-EBI, and NCBI), making the data of these databases effectively interchangeable (Karsch-Mizrachi et al., 2018). This type of integration initiative eliminates developer and researcher concerns regarding the “best” dataset for a sample and focuses on the importance and applicability of the analyses and outputs.

## Conclusion

Metagenomics is a promising tool for identifying and understanding antibiotic resistance mechanisms, using sequence- and function-based approaches. Notably, however, various analyses of antimicrobial resistance are strongly related to other aspects of the research being carried out, such as mutations, pathogens, metabolic pathways, and gene expression. Reviews analyzing antimicrobial resistance addressing these aspects are strongly recommended.

The most important considerations in a metagenomic resistome study are understanding the nature of the dataset being analyzed and the support that is available for its analysis. If one takes into account the large quantity of data and the complexity of the biological mechanisms involved in antibiotic resistance, it may be preferable to adopt reductionist approaches to decrease bias and increase the objectivity of analyses. It is important to emphasize that the costs of algorithms, computers, and analytical tools are decreasing; *in silico* predictions based on machine learning are thus becoming more common and have the potential to predict resistance outside databases. This will allow for the development of high-throughput data analysis approaches and the answering more complex questions regarding antimicrobial resistance.

## Author Contributions

SA and VA wrote the manuscript, as well as guided and reviewed the work. JP revised the writing and formulated the tables. All authors contributed to the article and approved the submitted version.

## Conflict of Interest

The authors declare that the research was conducted in the absence of any commercial or financial relationships that could be construed as a potential conflict of interest.

## Footnotes

1 https://www.ncbi.nlm.nih.gov/pathogens/beta-lactamase-data-resources/
